# Transcriptome changes caused by loss of WAGO-4 is remembered in wild type offspring

**DOI:** 10.17912/micropub.biology.001769

**Published:** 2025-08-25

**Authors:** Ann-Sophie Seistrup, Rene Ketting, Jan Schreier, Ida Isolehto

**Affiliations:** 1 Institute of Molecular Biology, Mainz, Germany; 2 EMBL, Monterotondo, Italy

## Abstract

The worm-specific Argonaute WAGO-4 has been shown to be involved in epigenetic memory in *C. elegans* , however, its mode of action remains unclear. Here, we use a *wago-4* deletion mutant to show that general changes to 22G-RNAs, the small RNA cofactor binding WAGO-4 , do not correlate with changes to mRNA levels. We also show that the function of WAGO-4 differs in L4 larvae and in adult worms, and, importantly, we show that mRNA misregulation caused by loss of WAGO-4 persists in wild-type offspring of deletion mutants for at least five generations.

**Figure 1. Transcriptome analysis of wago-4 mutants and wild-type siblings:**
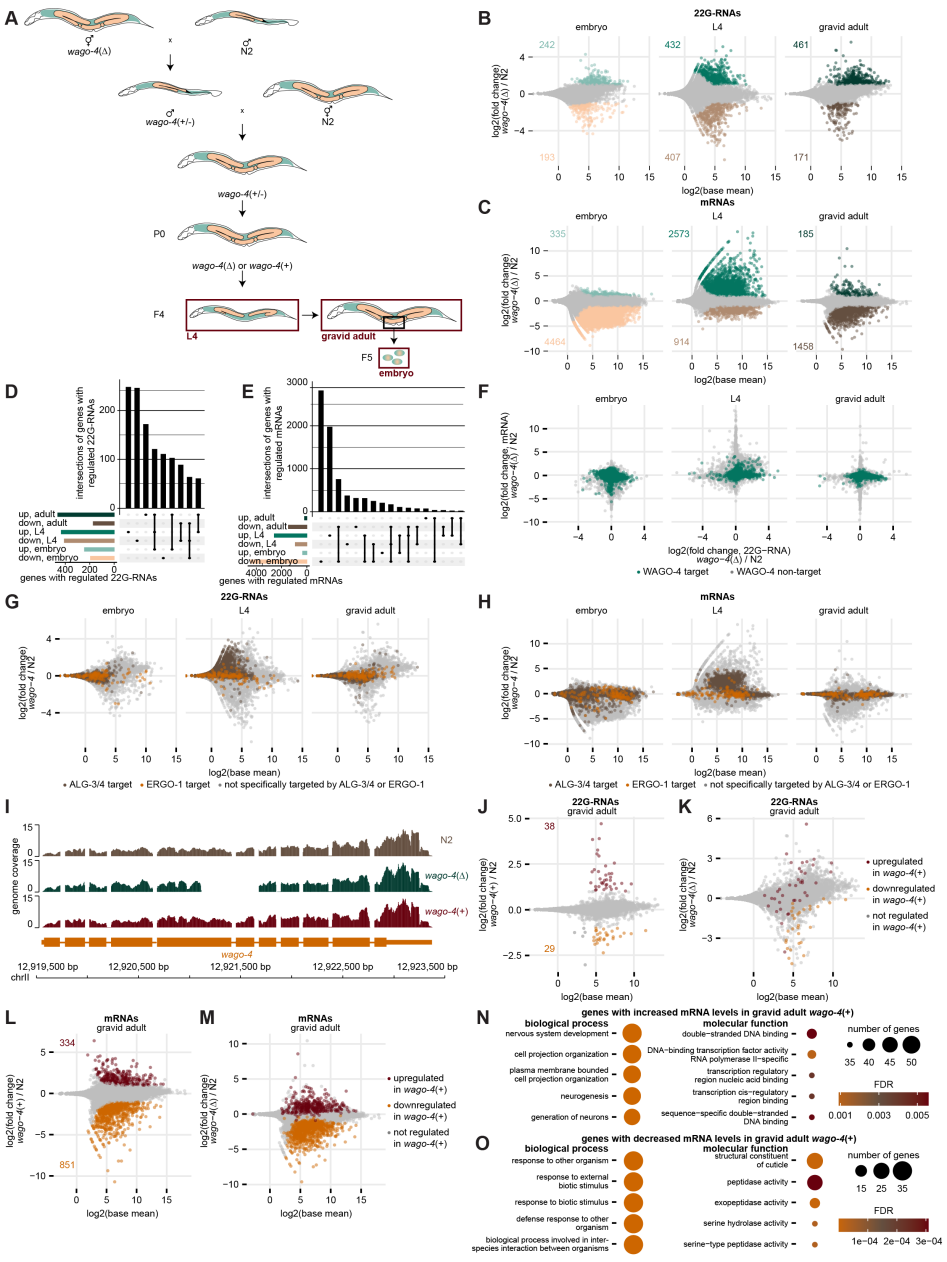
**Figure 1: A** Crossing scheme, red squares indicate generations harvested. **B** Scatterplots showing genes with affected 22G-RNAs in *wago-4 (* Δ) embryos, L4 larvae, and gravid adult worms. Each dot reflects one gene, typically with multiple 22G-RNA reads. Genes with significant changes (fold change > 2, p < 0.01) are indicated. **C** Scatterplots showing genes with affected mRNAs in *wago-4 (* Δ) embryos, L4 larvae, and gravid adult worms. Genes with significant changes (fold change > 2, p < 0.01) are indicated. **D** UpSet plot showing overlaps of genes with significantly changed 22G-RNAs. **E** UpSet plot showing overlaps of genes with significantly changed mRNAs. **F** Scatterplots comparing fold changes of mRNAs to fold changes of 22G-RNAs. Colours indicate genes determined to be WAGO-4 targets via RNA immunoprecipitation sequencing. **G** Scatterplots showing genes with affected 22G-RNAs in Δ *wago-4* mutants, as panel B. Colours indicate whether each gene has also been found as a target of ALG-3 /4 or ERGO-1 . Targets of both pathways are considered as targets of neither, and are shown in grey. **H** Scatterplots showing genes with affected mRNAs in Δ *wago-4* mutants, as panel C. Colours indicate whether each gene has also been found as a target of ALG-3 /4 or ERGO-1 . Targets of both pathways are considered as targets of neither, and are shown in grey. **I** Coverage tracks of wild-type ( N2 ), *wago-4 (* Δ), and *wago-4* (+) gravid adult worms along the *wago-4* gene. Only one replicate shown; all triplicates showed consistent patterns. **J** Scatterplot showing genes with changed 22G-RNAs in gravid adult restored wild-type versus original wild-type ( N2 ). Genes with significant changes (fold change > 2, p < 0.01) are indicated. **K** Scatterplot showing genes with changed 22G-RNAs in gravid adult *wago-4* versus original wild-type ( N2 ); same data points as panel B, ‘gravid adult'. Colours indicate significant hits in panel H. **L** Scatterplot showing genes with changed mRNAs in gravid adult restored wild-type versus original wild-type ( N2 ). Genes with significant changes (fold change > 2, p < 0.01) are indicated. **M** Scatterplot showing genes with changed mRNAs in gravid adult *wago-4* versus original wild-type ( N2 ) ; same data points as panel C, ‘gravid adult'. Colours indicate significant hits in panel J. **N** Bar plot showing the ten most numerous ‘molecular function' GO-terms and the ten most numerous ‘biological process' GO-terms all with adjusted p-values below 0.01 found in the genes with downregulated mRNA levels in the wild-type versus N2 . **O** Bar plot showing the ten most numerous ‘molecular function' GO-terms and the ten most numerous ‘biological process' GO-terms all with adjusted p-values below 0.01 found in the genes with upregulated mRNA levels in the wild-type versus N2 .

## Description

RNA interference (RNAi) has been shown to confer gene regulation in many organisms. It generally acts via suppression of an RNA, such as an mRNA, via a regulatory small RNA working in conjunction with an RNA-binding protein of the Argonaute family (Ketting, 2011). *C. elegans* expresses 19 different Argonaute proteins of which several belong to a worm-specific clade, the so-called WAGOs - worm-specific Argonaute proteins (Seroussi et al., 2023). While the importance of the high number of Argonaute proteins in *C. elegans* is still under study, it is known that only some of the WAGOs are involved in inheritance of RNAi (Schreier et al., 2022; Schreier et al., 2025; Xu et al., 2018).

WAGO-4 is germline-expressed, involved in transgenerational memory of exogenous RNAi, and has been shown to affect mRNA levels of genes targeted experimentally by RNAi (Schreier et al., 2025; Wan et al., 2018; Xu et al., 2018). WAGO-4 has also been shown to bind small RNAs against endogenous genes. These targets greatly overlap with the WAGO CSR-1 (Seroussi et al., 2023; Xu et al., 2018). However, it is unknown how WAGO-4 generally affects mRNA levels of these endogenous genes. Furthermore, the role of WAGO-4 has not previously been assessed throughout different life stages of *C. elegans* . We aimed to address both these questions.

Loss of a number of WAGOs causes a mortal germline (Mrt) phenotype, where worms gradually become sterile over time, presumably due to accumulation of errors caused by RNAi misfiring (Spracklin et al., 2017; Wan et al., 2018). In order to minimise such secondary effects, we outcrossed homozygous *wago-4 (* Δ) mutants to wild-type worms (hereafter referred to N2 ) and sequenced early (F4) homozygous offspring thereof ( **Figure 1A** ). We found that loss of WAGO-4 caused changes in 22G-RNA populations in both directions. It did so in embryos, L4 larvae, and adults ( **Figure 1B** ), and this was accompanied by changes in the levels of thousands of mRNAs in each life stage ( **Figure 1C** ). We noticed that mRNA changes differed in the different life stages, with the general tendency of reduction in adult worms and embryos, and more increase of mRNA levels in L4 larvae ( **Figure 1C** ). Most of the significantly changed 22G-RNAs were either specific to the life stage or regulated in the same direction in different life stages ( **Figure 1D** ). While most of the mRNAs undergoing regulation in the *wago-4 (* Δ) mutants were also specific to the life stage, several of the genes with reduced mRNA levels in the embryo showed significant upregulation in L4 larvae or gravid adult worms ( **Figure 1E** ), further emphasising the life stage specific effects of WAGO-4 . GO-term analysis revealed that the genes that had decreased mRNA levels in embryos and adult worms were largely involved in neurogenesis, whereas decreased mRNA levels in L4 larvae correlated to genes involved in defence response. In both embryos and L4 larvae, there was an increase of mRNAs of genes related to reproduction, gamete generation, and oogenesis. In L4, genes involved in phosphorylation/dephosphorylation furthermore showed higher expression. In the gravid adult, there was less consistency to the genes with enriched mRNA levels, with significant GO-terms ranging from metabolism of organic acids or lipids to defence response, but with 16 or fewer genes in each category.

Interestingly, changes to 22G-RNAs and mRNAs didn't show any correlation, positive or negative, neither for genes known to be targets of WAGO-4 nor for non-target genes ( **Figure 1F** ). The only exception for this was a small subset of 162 genes – 17 of which were previously defined as targets of WAGO-4 (Seroussi et al., 2023) – that showed increases of both 22G-RNAs and mRNAs in L4 larvae, given our significance thresholds (see Methods). This is opposite the negative correlation expected for silencing 22G-RNAs and may reflect a larger probability of generating 22G-RNAs against mRNAs with higher expression. The genes showing positive correlation were mostly related to protein phosphorylation/dephosphorylation, gamete formation and oogenesis. Overall, this data shows that *wago-4 (* Δ) mutants show wide-spread defects in 22G-RNA and mRNA levels, but also that no direct silencing effects can be detected through this bulk analysis. Moreover, we conclude that the effects of loss of WAGO-4 are life stage-dependant.

In order to determine which other Argonautes may be affected by the loss of WAGO-4 , we investigated how changes to 22G-RNAs and mRNAs correlated to targets of other Argonautes. We noticed a strong increase of both 22G-RNAs and mRNA levels coinciding with ALG-3 /4 target genes in L4 *wago-4 (* Δ *)* larvae ( **Figure 1G** and **H** ). ALG-3 /4 binds 26G-RNAs and is expressed in the spermatogenic gonad (Conine et al., 2010). Notably, targets of the Argonaute responsible for 26G-RNAs in the oogenic gonad and embryos, ERGO-1 (Han et al., 2009), were unchanged in all life stages ( **Figure 1G** and **H** ).

Along with F4 *wago-4 (* Δ) mutants, we also sequenced RNAs from F4 homozygous wild-type gravid adult worms from the same crosses in order to determine whether the misregulation caused by loss of WAGO-4 persisted in wild-type offspring. We confirmed via sequencing reads from our poly(A)-selected libraries ( **Figure 1I** ) that the *wago-4* gene was disrupted in the mutants and restored in the wild-type animals. Changes to the 22G-RNA landscape between these restored wild-types (hereafter referred to as *wago-4* (+)) and the original wild-type worms ( N2 ) were minor ( **Figure 1J** ). However, all 29 genes for which 22G-RNA levels were significantly depleted in the *wago-4* (+) worms coincided with genes tending towards reduction of 22G-RNAs in the deletion mutant ( **Figure 1K** ), indicating a small lingering effect of *wago-4* loss after it is restored. The genes with increased 22G-RNA levels in the *wago-4* (+) worms did not generally coincide with genes having increased 22G-RNA levels in *wago-4 (* Δ) mutants ( **Figure 1K** ), suggesting more stochasticity to positively changing 22G-RNAs. Interestingly, the mRNA levels in the *wago-4* (+) worms differed greatly from that of N2 worms ( **Figure 1L** ), and all mRNAs displaying significant differences in *wago-4* (+) had changed in the same direction in *wago-4 (* Δ) mutants ( **Figure 1M** ). Based on GO-term analysis, genes that remained downregulated in *wago-4* (+) worms were mainly related to neuronal processes and had functions related to transcriptional changes and DNA-binding ( **Figure 1N** ). Genes that remained upregulated were more involved in PTMs and defence responses ( **Figure 1O** ).

This study shows that changes in mRNA levels caused by the loss of WAGO-4 can persist for at least five generations in worms with restored WAGO-4 activity. This has implications for studies that analyse gene expression in strains derived from *wago-4* mutants. As we have not tested how long such changes can persist, we urge caution when doing such experiments with strains derived from *wago-4 mutants* , but possibly any *wago* mutant. The biological implications of this ‘memory' thus far remains unclear. Our GO-term analysis suggests that the animals possibly maintain a state of alert to pathogens, but a role for WAGO-4 in innate immunity has not yet been described. Finally, we note that WAGO-4 activity was restored via genetic crosses, raising the possibility that loci other than *wago-4* may contribute to the described effects.

## Methods

***C. elegans* strains and harvest**

Worms were cultured on nematode growth medium (NGM) plates (90 mm diameter) seeded with *Escherichia coli* OP50 in temperature-controlled incubators at 20°C. Worms were synchronized for life stage via bleaching and grown on egg plates, the preparation of which is described below. Worms were bleached a second time before being transferred to standard NGM plates, in the case of harvesting of L4 larvae or adult worms, or to egg plates when harvesting embryos. Harvesting was carried out using M9 buffer and aliquots were fast-frozen on dry ice before storage at -80°C.

Egg plates were made by mixing egg yolk with 50 ml LB medium per egg. After incubation at 65 °C for 2–3 hours, the mixture was cooled to room temperature before adding 10 ml OP50 culture per egg. About 10 ml was added to standard NGM plates (90 mm diameter) and incubated at room temperature. The next day, excess liquid was removed, and egg plates were incubated at room temperature for another 2 days.

**RNA extraction**

Harvested frozen worms were thawed on ice and 400 µl TRIzol LS Reagenz (10296010, Invitrogen) was added to the sample. The worm samples were then lysed by six cycles of freezing in liquid nitrogen followed by thawing at 37°C and vortexing. Embryo samples were lysed by being dropped into liquid nitrogen in a mortar, forming little balls that were subsequently pulverised using a pestle. Then 400 µl TRIzol LS Reagenz (10296010, Invitrogen) was added to the embryo samples. For all samples, liquid was transferred to a new tube and centrifuged for 5 min at 4°C and 21,000g. Supernatants were transferred into a fresh tube and RNA was extracted using Direct-Zol RNA Miniprep Kit (R2052, Zymo Research) according to the manufacturer's instructions and resuspended in nuclease-free water. RNA quality and quantity was assessed initially using the Bioanalyzer RNA 6000 Nano Kit (5067-1511, Agilent Technologies) and subsequently using the Qubit RNA BR Assay Kit (Q10210, Invitrogen). The same extracted RNA was used for sequencing of small RNAs and of mRNAs.

**Small RNA library preparation and sequencing**

NGS library prep was performed with NEXTflex Small RNA-Seq Kit V3 following Step A to Step G of Bioo Scientific`s standard protocol (V19.01) with a ligation of the 3' 4N Adenylated Adapter over night at 20°C. Previously to library prep, all samples were treated with RNA 5' Pyrophosphohydrolase (RppH, NEB). Libraries were prepared with a starting amount of 360 ng, addition of 1 µl NEXTflex tRNA/YRNA Blockers (BiooScientific) and amplified in 15 PCR cycles. Amplified libraries were purified by running an 8% TBE gel and size-selected for 144 – 163 nt. Libraries were profiled in a High Sensitivity DNA on a 2100 Bioanalyzer (Agilent technologies) and quantified using the Qubit dsDNA HS Assay Kit, in a Qubit 2.0 Fluorometer (Life technologies). All 23 samples were pooled in equimolar ratio, except for sample 6 which was pooled in a 1.2x ratio, and sequenced on a NextSeq 500/550 Flowcell, SR for 1x 75 cycles plus 7 cycles for the index read

**mRNA library preparation and sequencing**

NGS library prep was performed with Illumina's Stranded mRNA Prep Ligation Kit following Stranded mRNA Prep Ligation ReferenceGuide (June 2020) (Document # 1000000124518 v00). Libraries were prepared with a starting amount of 500 ng and amplified in 10 PCR cycles. For normalization, 1 µl of a 1:100 dilution of ERCC spike-ins (Ambion) was added. Libraries were profiled in a High Sensitivity DNA on a 2100 Bioanalyzer (Agilent technologies) and quantified using the Qubit dsDNA HS Assay Kit, in a Qubit 2.0 Fluorometer (Life technologies). All 23 samples were pooled in equimolar ratio and sequenced on a NextSeq500 Highoutput FC, SR for 1x 80 cycles plus 10 cycles for the index read and 1 dark cycle upfront.

**Read processing and mapping**

Raw sequenced reads from high-quality libraries, as assessed by FastQC, were processed using Cutadapt (Martin, 2011) for adapter removal (-a TGGAATTCTCGGGTGCCAAGG -O 5 -m 26 -M 48) and low-quality reads were filtered out using the FASTX-Toolkit (fastq_quality_filter, -q 20 -p 100 -Q 33). Unique molecule identifiers were used to remove PCR duplicates using a custom script and were subsequently removed using seqtk (trimfq-l 4 – b 4). Finally, in the case of small RNA-Seq, reads shorter than 15 nucleotides were removed using seqtk (seq -L 15). A custom GTF file was created by adding transposons retrieved from Wormbase ( PRJNA13758 .WS264) to the Ensembl reference WBcel235.84 and reads were aligned using bowtie (v.1.3.1) (Langmead et al., 2009)(--phred33-quals --tryhard --best --strata --chunkmbs 256 -v 2 -M 1) for small RNA-Seq or subread v.2.0.0 (Liao et al., 2013) featureCounts (--donotsort -t exon) for mRNA-Seq.

For sRNA-Seq, 22G-RNAs were extracted using a python script available at https://github.com/adomingues/filterReads and were defined as any read of length 20-23 nt with no bias at the 5'-position.

**Coverage tracks**

BigWig files were generated from mapped mRNA reads using deepTools v.3.5.5 bamCoverage (Ramírez et al., 2016) using default settings. These were visualised in the online tool Integrative Genomics Viewer (IGV) (Robinson et al., 2011). Tracks were downloaded as single vector graphics (SVG) files and recoloured using Adobe Illustrator.

**Differential analysis**

Reads were counted using htseq-count (v.2.0.2) (Anders et al., 2015) (-s no -m intersection-nonempty), and targets were determined using DeSeq2 (Love et al., 2014) in R, with a p-value cutoff of 0.01 and a cutoff for log2(fold change) of 1. For mRNA-Seq, reads mapping to ERCCs were used for normalization by resetting the size factors using estimateSizeFactors() on the data with only ERCC reads. All data was shrunk using lfcShrink() using the method ashr (Stephens, 2016).

Our data was compared to published RIP-Seq data of WAGO-4 , ALG-3 /4, and ERGO-1 from (Seroussi et al., 2023). Comparisons were carried out in R using the packages ggplot2 or UpsetR (Conway et al., 2017) for plotting. GO-term analyses were carried out using clusterProfiler (Yu et al., 2012).

## Reagents

**Table:** 

**Strain**	**Denotation**	**Acquisition**
N2 Bristol isolate	N2	*Caenorhabditis* Genetics Center (CGC)
*wago-4 ( tm1019 ) *	*wago-4 (* Δ)	*C. elegans* Deletion Mutant Consortium (2012)
*wago-4* (wild-type)	*wago-4* (+)	Generated via crossing of N2 and Δ *wago-4*

**Table:** 

**Chemical/Reagent**	**Company**
TRIzol LS Reagenz	Invitrogen
Direct-Zol RNA Miniprep Kit	Zymo Research
NEXTflex Small RNA-Seq Kit V3	BiooScientific
RNA 5' Pyrophosphohydrolase (RppH)	New England Biolabs (NEB)
NEXTflex tRNA/YRNA Blockers	BiooScientific
Illumina's Stranded mRNA Prep Ligation Kit	Illumina
ERCC Spike-Ins	Ambion

## Data Availability

Description: Read counts obtained from the sequencing experiments. Resource Type: Dataset. DOI: https://doi.org/10.22002/29vty-ptx66
